# Editorial: Metabolic Interactions Between Bacteria and Phytoplankton

**DOI:** 10.3389/fmicb.2018.00727

**Published:** 2018-04-10

**Authors:** Xavier Mayali

**Affiliations:** Lawrence Livermore National Laboratory (DOE), Livermore, CA, United States

**Keywords:** microalgae, bacteria, metabolites, 16S rRNA gene, roseobacter

## Introduction

Gone are the days when bacteria (and archaea) were largely ignored by oceanographers and limnologists. The study of microbes now dominates the aquatic sciences, as microbes do in activity and sometimes biomass, in most of earth's biomes (Whitman et al., 1998). Current efforts to better understand the impact of the human microbiome on our health (Cho and Blaser, 2012) underlie the major attitude change that we have had about the impact of microbial life on the rest of the world, from either unimportant or disease-causing to instrumental in maintaining a healthy ecosystem. In sunlit aquatic ecosystems (lakes, streams, estuaries, and the surface ocean), we know that bacteria processes on average 50% of the carbon fixed by photosynthesis (Azam and Malfatti, 2007), remineralizing CO_2_ and inorganic nutrients in the process. As such, the interactions between primary producing photoautotrophs (microalgae and cyanobacteria) and the secondary consuming and nutrient recycling heterotrophs (bacteria and archaea) are critical to understand ecosystem level processes, and it could be argued that these organisms should be studied together rather than in isolation.

Studies of bacterial-algal interactions, while becoming more common, have been undertaken for decades. In particular, some seminal papers paved the way for the recent papers that can utilize modern techniques to try to tackle old, still unanswered questions. For example, the term “phycosphere” was defined in 1972 (Bell and Mitchell, 1972) as the zone surrounding an algal cell analogous to the plant root rhizosphere, where the concentration of algal-derived molecules is thought to be higher than the bulk water. Due to methodological limitations, the phycosphere still has not been directly measured or quantified, though it has been modeled (Seymour et al., 2017), and this area is still ripe for study. Another seminal paper (Pomeroy, 1974) first suggested that bacteria were responsible for processing the majority of phytoplankton-fixed C in the ocean; this was later termed the microbial loop (Azam et al., 1983). Now decades later, bacteria are still not included in most biogeochemical models (Evans and Fasham, 2013), and in the rare case that they are, they are lumped into one black box, even if we know that not all bacteria are created equal. Clearly, more research on interactions between these two major guilds of organisms is needed.

This research topic presents 15 articles related to interactions between phytoplankton and bacteria, grouped into the following categories: (i) growth impact of bacteria on dinoflagellates, (ii) algicidal bacteria, (iii) algal-bacterial associations inferred from environmental surveys, (iv) chemical signaling, (v) competition for nitrogen and (vi) mesocosm manipulations. In addition, one final article reviews a type of metabolic modeling approach that may be useful to predict the impact of algal-bacterial interaction on ecosystem processes.

## Growth impact of bacteria on dinoflagellates

Phycologists long ago realized that removing bacteria from dinoflagellate laboratory cultures was difficult (Guillard and Keller, 1984) and many dinoflagellate species in fact cannot grow without bacteria. Such is the case with *Gymnodinium catenatum*, a harmful algal bloom causing species. Bolch et al. established laboratory batch co-cultures of this dinoflagellate with combinations of 1, 2, or 3 bacterial species and compared their growth, finding that bacteria can exert growth effects as strong as those of light and temperature. The 3 different bacterial strains, either alone or in the presence of others, exerted different growth effects on *G. catenatum*; in particular, a *Roseobacter* strain inhibited growth rate, led to lower maximum algal cell yield, and increased algal death rate. This effect was decreased but not completely eliminated by the presence of other bacteria, showing that bacterial competition can impact the interactions between algae and bacteria, as shown previously (Mayali and Doucette, 2002). In another study in this special topic, Cruz-Lopez examined the ability of bacteria to provide vitamins to the dinoflagellate *Lingulodinium polyedrum*. The authors first were able to render their algal culture axenic, then established bacterial enrichment cultures by adding 0.8 micron seawater filtrates. These cultures were maintained with or without additions of vitamins B_1_, B_7_, and B_12_. The authors showed that *L. polyedrum* is auxotrophic for B_1_ and B_12_, and that seawater bacteria can provide enough of those vitamins for long term culture growth. Interestingly, bacterial community taxonomy was not significantly different between cultures grown with and without added vitamins, suggesting that vitamin exchange is not a major factor in controlling which bacteria grow in the presence of the dinoflagellate.

## Algicidal bacteria

The effect of bacteria that kill algae (so-called algicidal bacteria) has been studied for decades (reviewed by Mayali and Azam, 2004). The large number of such studies is likely explained by, on the one hand, the need to mitigate harmful algal blooms (HABs) and understand the natural demise of these events in coastal zones, and on the other, by preventing algal pond crashes when algae are grown for aquaculture or other valuable products. Related to the latter, Ganuza et al. investigated the possibility of water treatment, through decreased *pH* for 15 min in the presence of acetate, to alleviate bacterial infection of the green alga *Chlorella* by the parasitic bacteria *Vampirovibrio*. The treatment was successful in prolonging algal cultivation, and the algicidal bacterium appears to be unable to build up immunity to this treatment (the impact on other bacteria was not characterized). Bagwell et al. also investigated the possibility of preventing *Vampirovibrio* infection of *Chlorella* cultures, in their case by inducing the production of bioactive small peptides and glycosides by *Chlorella* under iron limitation, which prevented *Vampirovibrio* infection. In another algal system, Mayers et al. investigated the interactions between the coccolithophore *Emiliania huxleyii* and a bacterial strain from the *Ruegeria* genus (Rhodobacteriaceae), finding that the bacterium is an opportunistic pathogen that kills certain *E. huxleyii* cell types only at higher temperatures.

## Associations inferred from environmental surveys

Moving from the laboratory to natural samples, three articles aim to link specific bacterial taxa with specific algal species. Sison-Mangus et al. examined the bacterial community structure at a coastal site over time through a number of blooms of the diatom genus *Pseudo-nitzschia*. They found that total bacterial diversity was lower when the bloom was dominated by *P. australis* that produce high amounts of the algal toxin domoic acid, and the communities were dominated by *Firmicutes* bacteria. Bloom samples from low toxin producing *P. fraudulenta* had higher diversity and were dominated by *Vibrio* bacteria. These results suggest that algal toxins potentially play a role in regulating bacterial community structure, although effects other than toxin production are also likely to be involved. At the same location, Farnelid et al. used flow cytometric cell sorting of photosynthetic picoeukaryotes followed by 16S rRNA sequencing to examine the bacterial communities physically-associated with these cells. These communities included both commonly-found free-living taxa, suggesting the eukaryotes potentially ingested these bacteria, as well as taxa not found free-living, suggesting symbiosis with specialized taxa (either intra or extracellular). Bunse et al. also aimed to correlate specific bacterial taxa with particular phytoplankton by sampling across the spring diatom bloom in the Baltic Sea. They found that numerically dominant bacterial taxa did not correlate well with algal bloom-related variables. On the other hand, several less-abundant bacterial taxa showed strong associations with algal bloom dynamics, including different *Bacteroidetes* taxa being associated with genetic subgroups of the dominant diatom.

## Chemical signaling

Indole 3 acetic acid (IAA) production from tryptophan has been previously identified as a metabolite involved in algal growth enhancement by bacteria (De-Bashan et al., 2008), and detected in the ocean (Amin et al., 2015; Segev et al., 2016). Labeeuw et al. investigated a different potential role of IAA as a signaling molecule among algal cells. They found that one type of axenic *E. huxleyi* cells produced IAA after tryptophan addition. *E. huxleyi* cultures co-incubated with a *Ruegeria* strain previously found to produce IAA from tryptophan were found to produce less IAA than the axenic cultures, which suggested to the authors that IAA is potentially not involved in bacterial-algal interactions between these organisms. This remains a controversial topic, as another possibility is that the IAA in the co-culture was incorporated more than in the algal monoculture. Regarding signaling molecules that inhibit growth, Harvey et al. identified the quorum sensing molecule 2-heptyl-4-quinolone (HHQ) as the compound responsible for growth inhibition of *E. huxleyi* when grown with the bacterium *Pseudoalteromonas piscicida*. This compound may be specific to *E. huxleyi* as it did not have any growth-inhibiting effects on two other algal strains, but the latter were not axenic cultures, suggesting bacteria-bacteria interactions may also play a role in this interaction.

## Competition for nitrogen

Two submissions to the research topic involved the examination of nitrogen cycling between microalgae and bacteria. First, Le Chevanton et al. used ammonium-limited chemostats to examine the impact of a strain of *Alteromonas* previously shown to increase the growth of *Dunaliella* in non-limiting batch cultures. Surprisingly, the presence of the bacterium led to a decrease in algal growth under these limiting conditions, and the bacterium did not remineralize organic nitrogen for algal uptake. This study demonstrates another example of an interaction between algae and bacteria that can change depending on the environment, going from mutualistic under ammonium replete to competitive under ammonium deplete conditions. In the same realm, Diner et al. examined competition for nitrate between another *Alteromonas* strain and the diatom *Phaeodactylum tricornutum*. They showed that under conditions without external inputs of organic carbon, the bacterium did not compete for nitrate and exhibited mutualistic effects, but with extra carbon in the form of pyruvate, it led to algal growth inhibition. Taking it one step further with genetic tests with mutants of both algae and bacteria, they showed that nitrate reductase-deficient bacteria did not inhibit algal growth in the presence of pyruvate and that wild-type bacteria could rescue growth of *P. tricornutum* nitrate reductase-deficient with nitrate as the sole N source, demonstrating direct mutualism: the algae provide organic C to the bacterium and the bacterium provides N back to the algae, in a form other than nitrate.

## Mesocosm manipulations

A type of study that is intermediate between laboratory and field involves bringing nature into the lab (or bringing the lab into nature) through the use of micro- or mesocosms. Russo et al. used a metaproteomic analysis to examine the succession of autotrophs (microalgae and cyanobacteria) and heterotrophic bacteria in freshwater mesocosms incubated under oligotrophic and eutrophic conditions. They found that *Bacteroidetes* expressed extracellular hydrolases and Ton-B dependent receptors to degrade and transport high molecular weight compounds in oligotrophic conditions, and that *Alpha*- and *Beta-proteobacteria* captured different substrates from algal exudate (carbohydrates and amino acids, respectively). They also found strong evidence of bacterial mixotrophy (either chemoautotrophy or photoheterotrophy), suggesting the division of algae and bacteria into autotrophs and heterotrophs may be outdated. Geng et al. followed the bacterial community structure of indoor microcosms associated with the saltwater microalga *Nannochloropsis salina* in the presence of antibiotics and signaling compounds. Using a network analysis of correlations, they found that a few bacterial taxa from the *Alteromonadaceae* and *Rhodobacteriaceae* were driving the community dynamics by being strongly associated with community “modules” that changed drastically across treatments. Further, they showed that tropodithietic acid, an antibiotic produced by members of the *Rhodobacteriaceae* (Wang et al., 2016), drastically changes bacterial community structure within a short timeframe, leading to the hypothesis that this and other compounds may be responsible for community structure dynamics.

## Conclusions

In order to better understand the impact of algal-bacterial interactions on processes of interest such as biogeochemical cycling, production of valuable or noxious compounds, and ecosystem impacts, it is becoming clear that experimental data must be used to better inform models that eventually hope to be predictive. One modeling approach discussed by Perez-Garcia et al., Stoichiometric Metabolic Network (SMN), can incorporate a variety of experimental data such as community structure, functional gene information (i.e., “omics data”) to mathematically represent cell biogeochemistry to quantify metabolic rates. Metabolic modeling of this type (e.g., Flux Balance Analysis, and similar) have been applied mostly to single species or very simple communities, but efforts will continue to investigate microbial interactions in more complex ecosystems, which may necessitate new modeling approaches.

In combination with new modeling approaches, experimental data, such as those published under this special topic, will need to continue to be collected if we are to take significant steps forward in our understanding of algal-bacterial interactions. The post-genomic era now enables cheap and fast generation of DNA, RNA, protein, and metabolite data, and new imaging methods are being developed to probe cell-cell interactions. I would argue that it is imperative to utilize these great tools in combination with well-designed experiments with environmentally-appropriate model systems or manipulations of natural samples incubated under relevant conditions, when specific effects can be directly tested. One challenge in this endeavor is that algal-bacterial interactions exist at the single cell scale, and an algal-dominated ecosystem is comprised of billions of single algal cells interacting with hundreds of different bacterial species. How can we determine which microscale algal-bacterial interactions have ecosystem-level consequences? What approaches can we take to apply what we learn from laboratory co-culture experiments to understand what occurs in the more complex natural environment? These are questions that future studies on algal-bacterial interactions should aim to address.

## Author contributions

The author confirms being the sole contributor of this work and approved it for publication.

### Conflict of interest statement

The author declares that the research was conducted in the absence of any commercial or financial relationships that could be construed as a potential conflict of interest.
